# Association Between UGT1A1 mRNA Expression and Cis-Acting Genetic Variants and Trans-Acting Transcriptional Regulators in Human Liver Samples

**DOI:** 10.3390/genes16080971

**Published:** 2025-08-18

**Authors:** Matthew J. Taylor, Joseph M. Collins, Abelardo D. Montalvo, Danxin Wang

**Affiliations:** Department of Pharmacotherapy and Translational Research, College of Pharmacy, Center for Pharmacogenomics and Precision Medicine, University of Florida, Gainesville, FL 32610, USA; matthewtaylor2@ufl.edu (M.J.T.); jcoll86@cop.ufl.edu (J.M.C.); admontalvo@ufl.edu (A.D.M.)

**Keywords:** UGT1A1, genetic polymorphisms, ancestry differences, gene expression, personalized medicine

## Abstract

**Background:** UDP-glucuronosyltransferase 1A1 (UGT1A1) metabolizes endogenous substances and pharmaceuticals. Genetic polymorphisms, particularly TA repeats in the UGT1A1 promoter TATA region (UGT1A1*28/*36/*37) and a nearby single-nucleotide polymorphism (SNP) rs887829, are associated with UGT1A1-related phenotypes and used as biomarkers for guiding drug therapy. However, these associations are inconsistent, especially in individuals of African ancestry. The objectives of this study are to investigate the association between UGT1A1 expression and its genetic variants in liver samples obtained from European American (EA, n = 119) and African American (AA, n = 138) donors and to clarify the function of genetic variants. **Methods:** The associations between UGT1A1 expression and genetic variants were tested using multiple linear regression analysis, and the transcriptional activities of genetic variants were tested using reporter gene assays. **Results:** Both rs887829 and UGT1A1*28/*37 showed similar associations with UGT1A1 expression in AA and EA samples. Reporter gene assays confirmed that UGT1A1*36 (5TA) had significantly higher activity than reference UGT1A1*1 (6TA), while UGT1A1*28 (7TA) and *37 (8TA) had lower activity. In contrast, rs887829 showed no direct effect on promoter activity, indicating that its association is likely caused by high LD with UGT1A1*28/*37. Additionally, we found that ancestral differences in associations with trans-acting regulators and combined genetic variants and TFs account for substantially higher total variability in UGT1A1 expression in EAs than in AAs (53% vs. 39%). **Conclusions:** Our findings reveal differences in UGT1A1 regulation between AA and EA populations and suggest that additional cis- and/or trans-acting factors regulating UGT1A1 expression remain to be discovered in individuals of African ancestry.

## 1. Introduction

The UDP-glucuronosyltransferases (UGTs) are a group of phase II drug-metabolizing enzymes that conjugate hydrophobic chemicals with a sugar moiety, facilitating their elimination via bile and urine [1]. This process plays an important role in the detoxification of endogenous and exogenous substrates. As the most studied member of the UGT superfamily, UGT1A1 is the sole enzyme responsible for the glucuronidation of bilirubin in humans [2] and metabolizes a broad range of pharmaceuticals such as irinotecan [3], dolutegravir [4], and belinostat [5], thereby playing crucial roles in both normal physiology and pharmacotherapy [6,7,8].

UGT1A1 genetic polymorphisms are associated with serum bilirubin levels and drug responses [6,8]. The most recognized polymorphisms occur in the gene’s promoter region, specifically within its TATA box (a thymine–adenine repeat sequence). This promoter TATA repeat polymorphism is characterized by either a gain or loss of TA repeats relative to the normal (or reference) A(TA)_6_TAA (6TA) sequence, resulting in variant alleles such as A(TA)_5_TAA (5TA), A(TA)_7_TAA (7TA), and A(TA)_8_TAA (8TA) denoted by UGT1A1*36, UGT1A1*28, and UGT1A1*37, respectively [8]. UGT1A1*28 and UGT1A1*37 are common reduced-function alleles associated with Gilbert’s syndrome [8], drug toxicities [6,9], and the discontinuation of drug treatments [10]. Consequently, the Clinical Pharmacogenetic Implementation Consortium (CPIC) and the Dutch Pharmacogenetics Working Group (DPWG) published clinical guidelines for utilizing UGT1A1*28 and *37 as biomarkers for atazanavir and irinotecan therapies [11,12].

In addition to promoter TA repeat polymorphisms, a single-nucleotide polymorphism (SNP) located 304 base pairs upstream of the UGT1A1 TATA box, known as rs887829 (or UGT1A*80), which is in near-perfect linkage disequilibrium (LD) with UGT1A*28 and UGT1A*37 combined in European populations [11]. This high LD between rs887829 and UGT1A1*28/*37 has led to the adoption of clinical guidelines that utilize rs887829 as a surrogate marker for guiding drug dosing regimens [11,13]. However, despite the clear associations between UGT1A1*28/*37 (or rs887829) and UGT1A1-related phenotypes in European populations, the results are inconsistent across different ancestral groups, especially among individuals of African ancestry. For example, while the UGT1A1 slow metabolizer genotype (rs887829 T/T) is associated with an increased risk of bilirubin-related discontinuation of atazanavir in White patients, this relationship was not observed in Black patients [14]. Similarly, UGT1A1*28 and rs887829 are associated with irinotecan-induced toxicity or dolutegravir serum concentrations in Asian and/or European populations [15,16,17,18] but are inconsistent in African populations [19,20,21].

In reporter gene assays, the 7TA and 8TA constructs have reduced transcriptional activity compared to the reference 6TA allele in HepG2 cells [22], indicating that they are the causal variants underlying UGT1A1*28 and *37. However, Matsui et al. demonstrated that the 7TA construct did not reduce reporter activity on its own and that instead, the combination of the 7TA and another SNP (−3275 or rs4124874) caused the reduced transcriptional activity of the UGT1A1*28 haplotype [23]. Genome-wide association studies (GWASs) have identified many other SNPs, including rs887829 as the top SNP associated with serum bilirubin levels in individuals of European and African descent [24,25], although direct experiments testing for a functional rs887829 effect have not been conducted. Thus, it remains unclear whether additional SNPs coinciding with the promoter TA repeat polymorphisms, such as rs887829, may contribute to the reduced expression of UGT1A1. Also, although associations between UGT1A1 genotypes and clinical phenotypes (e.g., serum bilirubin levels) have been reported in different populations [26], there are no reported direct measurements that link UGT1A1 genetic variants and UGT1A1 expression levels in different ancestral populations.

To fill these knowledge gaps, in this study, we investigated the association between the expression of UGT1A1 and promoter TA repeat polymorphisms or rs887829 in liver samples obtained from European American (EA) and African American (AA) donors. We used liver samples because UGT1A1 is primarily expressed in the liver. In addition, we employed reporter gene assays to clarify whether rs887829 influences UGT1A1 promoter activity in the context of the promoter TA repeat polymorphisms. We also explored the influences of trans-acting regulators on UGT1A1 expression in both populations. Our results showed ancestral differences in the regulation of UGT1A1 expression in liver samples and suggest the presence of additional cis- and/or trans-acting factors that regulate UGT1A1 expression in the African population.

## 2. Materials and Methods

### 2.1. Liver Samples

Liver specimens (total n = 258; EA = 119, AA = 138) were obtained from the Cooperative Human Tissue Network (CHTN, chtn.cancer.gov, accessed on 1 March 2025). All were indicated as histologically normal liver samples collected from cancer patients, most with metastatic colon cancer. The University of Florida Internal Review Committee approved the study (IRB201801313). There was no statistical difference in age (AA 56 ± 19 y vs. EA 60 ± 13 y, *p* > 0.05) or sex (AA 49% female vs. EA 55%, *p* > 0.05) between the sample groups (Table S1).

### 2.2. DNA and RNA Preparation

Genomic DNA was isolated using a Qiagen DNeasy kit (Qiagen, Germantown, MD, USA); total RNA was isolated using the Direct-zol RNA Miniprep kit (Zymo Research, Irvine, CA, USA). Complementary DNA (cDNA) was synthesized using the qScript Ultra Flex kit (Quantabio, Beverly, MA, USA) with gene-specific primers and oligo(dT) as described previously [27].

### 2.3. Gene Expression

UGT1A1 expression was measured using SYBR-Green qRT-PCR on a Quantabio Q instrument with gene-specific primers (Table S2). β-Actin served as the internal reference control. Relative expression was calculated as 2^ (Ct_β-actin—Ct_UGT1A1) × 10^6^. The data were log10-transformed and confirmed as normally distributed. The expression levels of transcription factors (TFs) were extracted from a previously published dataset [27,28].

### 2.4. Genotyping

The UGT1A1 TA repeats were genotyped using fluorescent fragment analysis on a SeqStudio Genetic Analyzer (Thermofisher, Waltham, MA, USA) as described previously [29]. PCR primers flanking the TATA box (Table S2) included a 5′-FAM label. Products were verified on 1% agarose gel and sized with GeneScan 600 LIZ (Thermofisher, Waltham, MA, USA); alleles *1 (6TA), *28 (7TA), *36 (5TA), and *37 (8TA) were identified via GeneMapper v5. The rs887829 genotypes were obtained from data generated by the University of Miami Genotyping Core using the Illumina GSAv3MD array (unpublished).

### 2.5. Multiple Linear Regression

We employed multiple linear regression to test the associations between UGT1A1 gene expression and the UGT1A1 promoter TA repeat polymorphisms or rs887829 genotype in the liver cohorts, adjusting for age, sex, and race. UGT1A1*28 and UGT1A1*37 were grouped as they have similar promoter activity reported previously [30]. UGT1A1*36 was grouped with UGT1A1*1 as reported in clinical guidelines [11]. We used an additive model and coded the promoter TA repeat polymorphism or rs887829 genotypes as 0, 1, or 2 to represent the number of variant alleles present. In addition, several TFs previously associated with UGT1A1 expression [28] were also tested. These include AHR, ARNT, ESR1, FOXA2, HNF4A, NFE2L2, NR1I2, NR1I3, PPARA, and RXRA, and a membrane-bound receptor, PGRMC1 (for convenience, we also refer to PGRMC1 as a TF). We used backward stepwise linear regression to select the most informative TFs to be included in the final model, with a stay threshold of *p* < 0.05. Model assumptions (homoscedasticity, normality, linearity, and multicollinearity) were verified. Post hoc power analysis was performed in R (pwr package, version 1.3) as described [29]. R 4.4 was used for all analyses.

### 2.6. Reporter Gene Constructs

Promoter fragments −350 to +150 (short construct) or −660 to +150 (long construct) relative to the UGT1A1 transcription start site were PCR-amplified and cloned into pGL3 (cloning sites XhoI and HindIII) using the In-Fusion HD kit (Takara Bio, San Jose, CA, USA). Primers are listed in Table S2. Plasmid DNA was prepared with ZymoPURE (Zymo Research, Irvine, CA, USA) and sequence-verified (Eton Bioscience, San Diego, CA, USA). The short constructs do not include rs887829, which is captured in the long constructs.

### 2.7. Site-Directed Mutagenesis

The UGT1A1*36 allele is in complete LD with a nearby SNP, rs34547608 (T > C) [30]. To test whether the effect of UGT1A1*36 is confounded by rs34547608, we converted the variant C allele to the reference T allele using site-directed mutagenesis with the Phusion Site-Directed Mutagenesis Kit (Thermo Scientific, Waltham, MA, USA). Site-directed mutagenesis primers are in Table S2. Following mutagenesis, plasmid DNA was purified using the ZymoPURE Plasmid Miniprep Kit (Zymo Research, Irvine, CA, USA). The modified plasmids were sequenced by Eton Bioscience (San Diego, CA, USA) to verify their fidelity.

### 2.8. Cell Culture, Transfection, and Reporter Gene Assays

HepG2 cells were cultured in DMEM supplemented with 10% fetal bovine serum and penicillin/streptomycin at 37 °C, 5% CO_2_ (ThermoFisher, Waltham, MA, USA). Cells were seeded in 24-well plates one day before transfection to achieve 70–90% confluence. Transfection was performed using Lipofectamine 3000 (Thermo Fisher Scientific, Waltham, MA, USA) with 0.5 µg reporter plasmid and 50 ng Renilla control (pGL4.74; Promega, Madison, WI, USA), and cells were incubated for 24 h before the assay. Luciferase activity was measured using the Dual-Luciferase Reporter Assay System (Promega, Madison, WI, USA), according to the manufacturer’s instructions. Luminescence was quantified using a Varioskan LUX multimode microplate reader (Thermo Fisher Scientific, Waltham, MA, USA). Firefly luciferase activity was normalized to Renilla activity to account for variability in transfection efficiency. All experiments were performed in triplicate, and the results are presented as the mean ± standard deviation of at least two independent experiments.

### 2.9. Statistical Analysis

Data are shown as mean ± SD. Statistical analyses were performed using R4.4.

## 3. Results

### 3.1. The Allele Frequencies of UGT1A1 *28, *37, *36, and rs887829 in Liver Samples

The minor allele frequencies (MAFs) of the UGT1A1 variants tested are shown in Table 1. The MAF for UGT1A1*28 was higher in AA (37%) than in EA (32%), with an overall frequency of 35%, consistent with the NCBI database (ncbi.nlm.nih.gov). As expected, UGT1A1*37 and UGT1A1*36 are rare in EAs but are more commonly occurring in AAs. rs887829, which is in high LD with UGT1A1*28 and *37, also had a higher MAF in AAs (45%) than in EAs (33%).

### 3.2. The Linkage Disequilibrium Between UGT1A1*28, *37, *36, and rs887829

We assessed LD between rs887829 and the UGT1A1 alleles (*28, *37, and *36) in our liver cohort (Table 2). In EA samples, UGT1A1*28 and rs887829 are in strong LD (D′ = 0.98, r^2^ = 0.93). In AA samples, the D′ remains high (0.98) but the r^2^ is lower (0.77), indicating a weaker correlation between UGT1A1*28 and rs887829 allele frequencies in AAs. These LD results closely match those previously reported [13]. In contrast, UGT1A*37 had high D’ values in both AAs and EAs (AA: 0.99; EA: 0.99) but notably lower r^2^ values (AA: 0.08; EA: 0.02) due to the differences in the MAF between *37 (rare) and rs887829 (frequent). Because rs887829 co-occurs with both *28 and *37, we combined the *28 and *37 individuals into a single group. As expected, the combination of UGT1A1*28/*37 showed a higher LD with rs887829 than UGT1A1*28 or *37 alone (Table 2). The UGT1A1*36 allele exhibited moderate D’ (0.71 in both ancestries) and low r^2^ values (0.03) with rs887829, indicating lower LD between these two genetic polymorphisms.

### 3.3. Testing the Association Between UGT1A1 Expression and the Promoter TA Repeat Polymorphism or rs887829 in the Liver Samples

The association between UGT1A1 expression and UGT1A1 variants, after adjusting for age, sex, and race, is shown in Table 3. In the AA and EA combined cohort, UGT1A1*28/*37 and rs887829 were significantly associated with UGT1A1 expression, while age, sex, and race were not. When AA and EA samples were analyzed separately, rs887829 and UGT1A1*28/*37 showed comparable associations in both ancestries.

**Figure 1:**
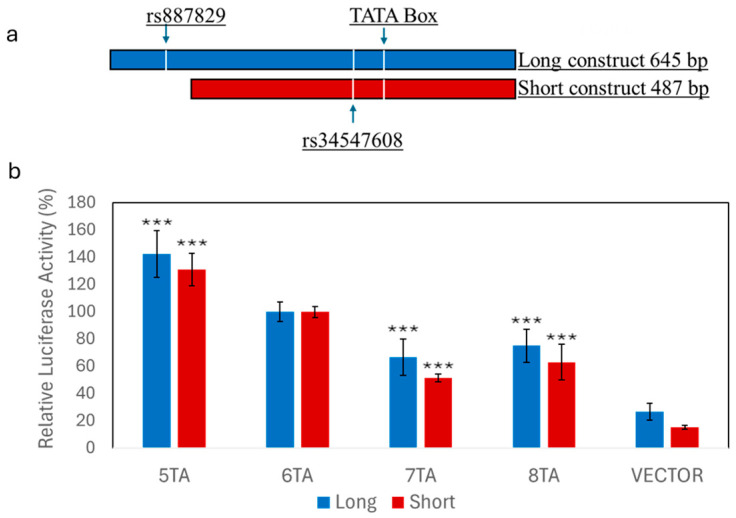
Luciferase reporter assay testing the effect of the UGT1A1 TA promoter repeats polymorphisms. (**a**) Diagram of the long (645 bp, blue) and short (487 bp, red) UGT1A1 promoter–luciferase constructs used in this study. Locations of TA-repeat/TATA box and the flanking SNPs rs887829 and rs34547608 are shown. (**b**) Relative luciferase activities of TA repeat polymorphisms using both long and short constructs. Luciferase activities of constructs with different TA repeats were normalized to the activity of 6TA, set at 100%. Compared to 6TA, *** *p* < 0.001, ANOVA with Tukey post hoc test.

**Figure 2:**
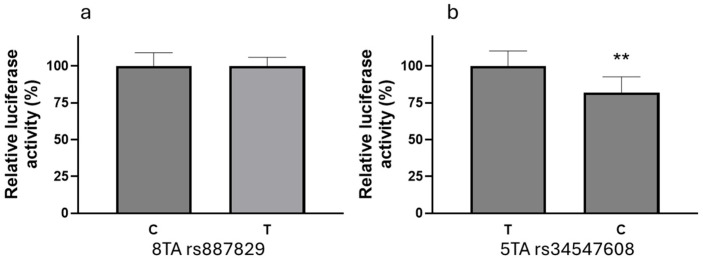
The effect of rs887829 and rs34547608 on reporter gene activity. (**a**) Comparison of the relative luciferase activities of long UGT1A1 promoter constructs carrying the 8TA repeat and the different rs887829 genotypes (reference C vs. variant T). (**b**) Comparison of the relative luciferase activities of the short UGT1A1 promoter constructs carrying the 5TA repeat and the different rs34547608 genotypes (reference T vs. variant C). ** *p* < 0.01, *t*-test.

We then tested whether rs887829 or UGT1A1*28/*37 directly affected UGT1A1 promoter activity using reporter gene assays in HepG2 cells. Two constructs with different lengths were evaluated; the short constructs only contained the region surrounding the TA repeat polymorphism, while the long constructs also were extended to include rs887829 (Figure 1a). There was an inverse relationship between the number of TA repeats in a construct and the reporter gene activities, with 5TA (*36) > 6TA (*1) > 7TA (*28) = 8TA (*37) (Figure 1b), consistent with a previous report [30]. The results were similar for the short and long constructs, indicating that rs887829 may not contribute to the reduced activity of 7TA or 8TA. To test this further, we compared the reporter gene activities between the rs887829 C and T alleles using the 8TA constructs. There were no differences in luciferase activity between rs887829 C or T alleles (Figure 2a), indicating that rs887829 does not affect UGT1A1 promoter activity.

Constructs carrying the 5TA had increased reporter gene activity compared to the 6TA (Figure 1b). The 5TA constructs also contained rs34547608 (Figure 1a), which is in complete LD with 5TA. To test whether rs34547608 affects promoter activity, we converted the variant rs34547608 C allele to the reference T allele through site-directed mutagenesis in 5TA short constructs. The variant C allele, normally co-occurs with 5TA, showed a small but significant decrease in luciferase activity compared to the reference T allele (Figure 2b). This result indicates that rs34547608 variant C allele may attenuate the increased activity of the 5TA, but the overall activity of 5TA is still higher than 6TA (Figure 1b).

In prior association models, we grouped UGT1A1*36 with UGT1A1*1 (Table 3), as conducted in previous association studies [31,32]. Because UGT1A1*36 (5TA) increases promoter activity (Figure 1b) and is common in AAs, we tested whether UGT1A1*36 has an independent effect on UGT1A1 expression. We included UGT1A1*36 as an independent variable alongside UGT1A1*28/*37 in the regression model. In agreement with its effect in the reporter gene assays, UGT1A1*36 showed a positive association with UGT1A1 expression (β = 0.145), although this association was not statistically significant (*p* = 0.380) (Table 3). The lack of significance was likely due to the low allele frequency of UGT1A1*36 and insufficient statistical power (~10% power, Table S3). Furthermore, including UGT1A1*36 as an independent variable in our model did not change the association between UGT1A1*28/*37 and UGT1A1 expression.

### 3.4. The Inclusion of Trans-Acting TFs Improves the Association Between Genotypes and UGT1A1 Expression in Liver Samples

The expression of the liver drug-metabolizing enzymes, including UGTs, is known to be regulated by numerous liver-enriched TFs [33,34,35,36,37,38,39,40], and our previous report identified several TFs to be associated with the expression of the UGTs in liver samples [28]. Thus, we tested how the inclusion of these TFs influenced the relationship between UGT1A1 expression and UGT1A1 genotypes. TFs tested included AHR, ARNT, ESR1, FOXA2, HNF4A, NFE2L2, NR1I2, NR1I3, PPARA, RXRA, and PGRMC1 [33,34,35,36,37,38,39,40]. We employed a backward stepwise linear regression approach to select the optimal set of variables to include in the model, ensuring that age, sex, and UGT1A1*28/*37 or rs887829 were maintained. The analysis was performed separately for AA and EA samples. As expected, including TFs in the model explained substantially more total variability in UGT1A1 expression for both AAs and EAs. We observed that the effect was more pronounced for EAs than for AAs, with total adjusted R-squared values increasing by ~37% in EA samples compared to ~28% in the AA samples (Table 3 and Table 4) and the overall total variability explained was higher in EAs than in AAs (R^2^ 53% vs. 39%). Notably, PGRMC1 emerged as the only significant factor in AAs, while seven TFs (ARNT was the top) were significant in EAs, indicating differences in the regulation of UGT1A1 between AA and EA liver samples. Notably, after adjusting for these TFs, the associations between the genotypes (UGT1A1*28/*37 or rs887829) and UGT1A1 expression became more significant for both sample groups (Table 4), but the improvements were more pronounced for EAs than AAs (Table 3 and Table 4).

## 4. Discussion

In this study, we investigated the association between UGT1A1 gene expression and UGT1A1 genetic variants (UGT1A1*28/*37 and rs887829) and TFs in liver samples and compared the differences between African and European ancestries. We also tested the regulatory function of the UGT1A1 repeat polymorphism and rs887829 using reporter gene assays. Our results confirmed the regulatory functions of UGT1A1*28/*37, but not rs887929. Additionally, our results revealed a substantial difference in the involvement of TFs in UGT1A1 expression between AAs and EAs.

GWASs have shown rs887829 as a top SNP associated with UGT1A1-related phenotypes in both African and European ancestry [13,24,25]. However, the regulatory function of rs887929 is unclear, and these studies did not compare the association between rs887829 and UGT1A1 *28/*37 with UGT1A1-related phenotypes because the promoter TAs are typically not included in genome-wide genotyping arrays. Here, we compared the associations of rs887829 and UGT1A1*28/*37 with UGT1A1 mRNA expression in AA and EA liver samples. We found similar associations between these two variants in both ancestries. Reporter gene assays revealed reduced activity of UGT1A1*28 and *37, consistent with previously reported findings [30], while no differential effect was observed for the rs887829 genotype (Figure 2a). This result supports the notion that UGT1A1*28/*37 are the causal variants for the observed associations, and the association observed for rs887829 is likely due to its high LD with UGT1A1*28/*37 (Table 2).

UGT1A1*36 (5TA) showed increased luciferase activity in reporter gene assays (Figure 1b). UGT1A1*36 is typically grouped with UGT1A1*1, and its independent effects have not been tested in previous association studies [31,32]. Our results showed a trend toward an association between UGT1A1*36 and increased UGT1A1 expression; however, we were underpowered to statistically confirm the predicted change. Thus, UGT1A1*36 may enhance UGT1A1 expression and activity, warranting further investigation in a larger cohort.

Comparison of the EA and AA liver samples showed that different trans-acting TFs are associated with UGT1A1 expression depending on sample ancestry. In AA samples, PGRMC1 emerged as the only significant factor associated with UGT1A1 expression (Table 4). In contrast, in the EA samples, several TFs, including PGRMC1, were significant, with ARNT as the top TF. Therefore, UGT1A1 expression may be controlled through distinct regulatory networks across populations, further contributing to the observed phenotypic differences between the ancestries. After adjusting for the TFs, the associations between UGT1A1*28/*37 or rs887829 and UGT1A1 expression became stronger in both AA and EA cohorts (Table 4), but this improvement was slightly more pronounced in EAs than in AAs. Additionally, the total variability in UGT1A1 expression explained by both cis-acting genetic variants and trans-acting TFs was also higher for EAs than AAs (Table 4), suggesting the presence of additional regulators yet to be identified in AAs.

PGRMC1 is a membrane-bound receptor localized at various subcellular compartments and is known to be involved in signaling cascades [41]. PGRMC1 forms multi-protein complexes that can directly bind to heme and can form protein complexes with the CYPs, functioning in a variety of processes, including cholesterol synthesis and drug metabolism [42,43]. We previously found a strong association between PGRMC1 and expression of CYP3A4, but targeted up- or down-regulation of PGRMC1 did not affect CYP3A4 expression [44], indicating that PGRMC1 may not be a direct CYP3A4 regulator. Similarly, we detected a strong correlation between the expression of PGRMC1 and UGT1A1 in liver RNA-Seq data from GTEx and our liver cohort [28]. These previous results prompted us to test it here, where we discovered its strong association with UGT1A1 expression in the AA samples. The connection between PGRMC1 expression and UGT1A1 expression is intriguing, as both play roles in heme metabolism. UGT1A1 is responsible for the metabolism and elimination of bilirubin, a by-product of heme catabolism [45]. PGRMC1 is a potential heme chaperone that binds to and regulates its synthesis, and it may act as a heme sensor [46]. This close functional relationship between UGT1A1 and PGRMC1 in the heme pathway may provide a plausible biological explanation for their co-expression [47]. Since PGRMC1 is known to play a role in signaling, it seems plausible that it may act in a regulatory loop that connects heme catabolism to bilirubin elimination. However, it remains unclear why the association between PGRMC1 and UGT1A1 expression is stronger in AAs than in EAs, which warrants further investigation.

The UGT1A1 promoter repeats and rs887829 are currently used to predict UGT1A1 activity [11,13]. Our findings suggest that pharmacogenetic guidelines may need to be tailored to specific ancestral groups, as the predictive markers and regulatory mechanisms underlying UGT1A1 expression differ across populations. The persistent 14% gap in total variability explained between AAs and EAs after accounting for known variants and TFs may explain the previously observed inconsistent associations between UGT1A1 variants and UGT1A1-related phenotypes in African populations [14,19,20,21]. This underscores the urgent need to identify additional cis- or trans-regulatory factors in African populations to achieve equitable pharmacogenetic prediction.

A possible limitation of this study is that we did not measure UGT1A1 activity, which ultimately determines clinical phenotypes. However, the focus of this study is to understand the transcriptional regulation of UGT1A1 by cis-acting genetic variants and trans-acting TFs, measuring UGT1A1 mRNA expression acts as a direct readout. Understanding factors that regulate UGT1A1 post-transcriptional, translational, and post-translational processes, which determine UGT1A1 protein and activity, is an interesting future direction. Also, we only assayed two clinically relevant promoter polymorphisms without screening for additional variants, and we only tested a selected set of liver-enriched TFs. Future studies will focus on identifying additional causal variants that regulate UGT1A1 expression using GWAS-based and functional genomics approaches. Additionally, investigation of the differential expression and function of TFs between ancestral groups could provide deeper insights into the mechanisms underlying UGT1A1 regulation. Ultimately, deciphering these intricate regulatory networks will be crucial for refining our understanding of UGT1A1 expression and will lead to the development of improved UGT1A1 biomarkers for personalized drug therapy that are effective for all populations.

## Figures and Tables

**Table 1:** Minor allele frequencies of UGT1A1*28, *37, *36, and rs887829.

	AA + EA	AA	EA
Variants	n = 257	n = 138	n = 119
UGT1A1*28	0.35	0.37	0.32
UGT1A1*37	0.03	0.05	0.008
UGT1A1*36	0.05	0.07	0.013
rs887829 (*80)	0.39	0.45	0.33

**Table 2:** The LD between rs887829 and UGT1A1 promoter repeat polymorphisms in EA and AA cohorts.

TA Repeats		AA		EA
	D’	r2	D’	r^2^
UGT1A1*28	0.99	0.77	0.98	0.93
UGT1A1*37	0.99	0.08	0.99	0.02
UGG1A1*36	0.68	0.03	0.71	0.03
UGT1A1*28/*37	0.99	0.94	0.99	0.98

**Table 3:** Association between UGT1A1 expression and promoter TA repeats or rs887829 in liver samples obtained from AA and EA donors.

Genetic Variants	AA + EA			AA			EA		
	Beta Estimate	*p*-Value	r^2^	Beta Estimate	*p*-Value	r^2^	Beta Estimate	*p*-Value	r^2^
UGT1A1*28/*37 ^a^	−0.33	1.65 × 10^−8^	0.13	−0.336	4.18 × 10^−5^	0.12	−0.343	2.62 × 10^−5^	0.16
rs887829 ^a^	−0.325	2.16 × 10^−8^	0.12	−0.343	2.93 × 10^−5^	0.13	−0.33	4.12 × 10^−5^	0.15
UGT1A1*36 ^a,b^	ND	ND	ND	0.145	3.802 × 10^−1^	0.04	ND	ND	ND

^a^ Age, sex, and race (for AA + EA only) were included as co-variates. ^b^ After controlling for UGT1A1*28/*37 genotype.

**Table 4:** The association between UGT1A1 expression and promoter TA repeats or rs887829 in AA and EA samples after adjusting for the expression levels of TFs.

Race	Independent Variables	UGT1A1*28/*37 Genotype			rs887829 Genotype		
		Beta Estimate	*p* Value	Partial R^2^	Beta Estimate	*p* Value	Partial R^2^
**AA**	*28/*37	−0.3	1.30 × 10^−5^	0.134	−0.312	6.16 × 10^−6^	0.142
	Age	0.002	0.376	0.006	0.002	0.32	0.017
	Sex	0.065	0.468	0.004	0.08	0.371	0.014
	PGRMC1	0.937	2.57 × 10^−12^	0.309	0.94	1.74 × 10^−12^	0.316
		Total adjusted R^2^ = 39%			Total adjusted R^2^ = 39%		
**EA**	*28/*37	−0.341	9.29 × 10^−8^	0.233	−0.334	1.16 × 10^−7^	0.23
	age	−0.002	0.626	0.002	−0.001	0.664	0.002
	sex	−0.125	0.129	0.021	−0.13	0.115	0.023
	ARNT	−1.517	3.34 × 10^−6^	0.182	−1.554	2.08 × 10^−6^	0.189
	AHR	1.045	0.0001	0.13	1.065	8.66 × 10^−5^	0.134
	PPARA	0.677	0.0002	0.119	0.667	0.0002	0.116
	ESR1	0.345	0.001	0.096	0.353	0.001	0.1
	PGRMC1	0.632	0.004	0.076	0.633	0.004	0.076
	NFE2L2	−0.54	0.02	0.049	−0.562	0.016	0.053
	NR1I2	−0.424	0.0456	0.036	−0.401	0.059	0.033
		Total adjusted R^2^ = 52%			Total adjusted R^2^ = 53%		

## Data Availability

The original contributions presented in this study are included in the article/supplementary material. Further inquiries can be directed to the corresponding author(s).

## Supplementary Materials

The following supporting information can be downloaded at https://www.mdpi.com/article/10.3390/genes16080971/s1; Table S1. Demographic information of liver donors; Table S2. Sequence of primers; Table S3. Post Hoc power analysis for variants in AA and EA cohorts.
