# Supplementary material for: Association Between UGT1A1 mRNA Expression and Cis-Acting Genetic Variants and Trans-Acting Transcriptional Regulators in Human Liver Samples
Source: Genes (Basel). 2025 Aug 18;16(8):971. doi: 10.3390/genes16080971 (PMC12385207; doi:10.3390/genes16080971)
Supplement: Supplementary file 1 [file genes-16-00971-s001.zip › genes-3790245-supplementary.pdf]

Table S1. Demographic information of liver donors. AA=African Americans; EA=European Americans

	AA + EA	AA	EA	p value
Number (n)	257	138	119	
Age, years, median (range)	58 (0-97)	56 (0-97)	60 (14-83)	0.068
Female, n (%)	133 (48)	68 (49)	65 (55)	0.39

Table S2. Sequence of primers

PCR primers	Primer sequence	PCR conditions
Short Construct	F: ACATAGTCGTCCTTCTTCCTCTCTGGTAAC	PrimeSTAR 2X PCR Mix.
	R: CAGCTGCTGGATGGCCCC	30 Cycles of [95 °C for 10 sec, 65 °C for 5 sec, 72 °C 7 sec]
Long Construct	F: GATACAAGTGAGCAGGCAGTACCGG	PrimeSTAR 2X PCR Mix.
	R: R: CAGCTGCTGGATGGCCCC	30 Cycles of [95 °C for 10 sec, 65 °C for 5 sec, 72 °C 7 sec]
rs34547608 SDM	F: ACAGCTTTTTATAGTCACGTGACACAGTC	Initial denaturation at 98°C for 30 sec30 Cycles of [Denature at 98°C for 10 sec, 67.6°C for 20 sec, 72°C for 2:30 min]Final extension at 72°C for 5 min
	R: CAGTCCACAAAGGTAGCAGGGAG	
TA repeats Genotyping	F: FAM-CACGTGACACAGTCAAACATTAACTTG	JumpStart 2 x PCR mix. Initial denaturation at 95C for 3 min. 40 cycles of [95C for 15 sec, 60C for 30 sec, 72C for 1 min], 72C for 10 min.
	R: CAACAGTATCTTCCCAGCATGGG	
UGT1A1 gene expression	F: TCATGCCCAATATGGTTTTTG	
	R: AATGTAGGCTTCAAATTCCTGG	

Table S3. Post hoc power analysis for variants in AA and EA cohorts.

	Variant	Population	R Squared	F Squared	Power
Without TFs	UGT1A1*28/*37	Combined	0.052	0.054	0.961
	UGT1A1*28/*37	AA	0.055	0.058	0.805
	UGT1A1*28/*37	EA	0.053	0.056	0.723
	rs887829	Combined	0.05	0.053	0.957
	rs887829	AA	0.058	0.062	0.827
	rs887829	EA	0.048	0.051	0.682
	UGT1A1*36	AA	0.003	0.003	0.095
With TFs	UGT1A1*28/*37	Combined	0.045	0.047	0.935
	UGT1A1*28/*37	AA	0.044	0.046	0.705
	UGT1A1*28/*37	EA	0.052	0.055	0.719
	rs887829	Combined	0.045	0.048	0.936
	rs887829	AA	0.048	0.051	0.747
	rs887829	EA	0.049	0.052	0.683
	UGT1A1*36	AA	0.003	0.003	0.106
